# Solving the inverse problem for coarse-mode aerosol particle morphology with digital holography

**DOI:** 10.1038/s41598-017-09957-w

**Published:** 2017-08-24

**Authors:** Matthew J. Berg, Yuli W. Heinson, Osku Kemppinen, Stephen Holler

**Affiliations:** 1 Department of Physics, Kansas State University, 1228 N. 17th St., Manhattan, KS 66506 USA; 2 Department of Energy, Environmental & Chemical Engineering, Washington University in Saint Louis, One Brookings Drive, Box 1180, Saint Louis, MO 63130 USA; 3 Department of Physics & Engineering Physics, Fordham University, 441 E. Fordham Rd., Bronx, NY 10458 USA

## Abstract

Coarse mode atmospheric aerosol particles are abundant in agricultural, desert, and urban environments. Accurate characterisation of these particles’ morphology is an important need in scientific and applied contexts, especially to advance our understanding for how such aerosols influence solar radiative forcing of the atmosphere. Elastic light scattering is a standard method to study aerosol particles in a contact-free manner, wherein measured scattering patterns are interpreted to infer particle morphology. Due in part to the absence of wave-phase information in these measurements, the inference is not unique, a difficulty generally known as the inverse problem. An alternative approach is digital holography where wave-phase information is encoded in the measurements. We show that digital holography and spatial filtering can solve the inverse problem for free-flowing aerosol particles in the sense that a measured scattering pattern can be uniquely associated with the particle size, shape, and orientation producing it.

## Introduction

Aerosols, whether anthropogenic or natural, are ubiquitous in the environment and there is need to accurately characterize their physical form. For example, a key component to understanding climate change is to quantify how aerosols contribute to the radiative forcing of the atmosphere. Recent studies^1–3^ find that the estimated aerosol forcing is comparable in magnitude to all other factors, yet, the uncertainty is nearly as large as the forcing value itself ^3–5^. One source of this uncertainty is the use of unrealistically simple particle shapes in climate models, which is partly due to the lack of accurate *in*-*situ* observations of the particles present^2, 5–8^. The need for such observations is especially relevant for coarse-mode aerosols (CMAs), which are complex in morphology and include airborne mineral dust^8^ (MD), bioaerosols of pollens and plant fragments^9^, large combustion particles from wildfires^10^, and volcanic ash^11^. Such particles are ~1–100 μm in size and are important to study because, e.g., they can dominate the aerosol mass-distribution in desert and agricultural regions, and can be transported thousands of kilometres^9, 11–14^. In particular, it remains unclear whether MD has a net global heating or cooling effect^3, 15^. The size-distributions of these aerosols continue to be poorly understood, and while some conventional microscopy^16^ has been done, a systematic *quantitative* description of particle morphology is lacking^13^. These issues underpin the need for methods to ascertain CMA particle morphology without measurement-based shape-distortions, as is the case, e.g., when particles are collected on substrates for later analysis. Addressing this need is also important because the accuracy of remote-sensing retrieval efforts, in part, rely on the particle morphologies assumed, and could thus benefit greatly from such characterisation abilities^17–21^.

Perhaps the most suitable technique available for aerosol studies is optical light-scattering due to its contact-free and rapid nature^22, 23^. A particle’s scattering pattern depends sensitively on its morphology, composition, and orientation, so proper interpretation of a measured pattern can, in principle, be useful for particle characterisation^23–28^. Unfortunately, no unambiguous relationship between a measured pattern and the particle characteristics is available–a difficulty known as the “inverse problem”^23^. Precisely what the “proper interpretation” of a pattern ought to be remains an active area of debate^29^, and indeed, no method has demonstrated the ability to *confidently* characterize arbitrary particles *in situ* ^30^. Coarse-mode particles are particularly challenging in this regard because their large (optical) size, nonspherical shape, and inhomogeneous composition yield patterns rich with complexity^29, 31, 32^.

An alternative to light scattering is to simply image particles directly with, e.g., a telemicroscope. This approach is feasible if the resolution of the imaging system is sufficient for the particle size under study, and thus, is applicable for micron-sized and larger particles. In such cases, the depth of focus is then restricted to micrometers as well. An instrument based upon this apprach must then catch particles in electrodynamic^33–35^ or optical traps^36, 37^, or flow particles in a stream with trajectories confined to micrometers^30, 38^. Not only is it challenging^30^ to control aerosol-particle flow on such length scales, but a blurred image resulting from a particle that fails to pass through the focal region cannot be re-focused since the particle is no longer present. Moreover, from a radiative forcing perspective, it is also desirable to know the particle’s scattering pattern, which is not available in a direct-imaging approach. As such, one must then simulate the pattern from the image using numerical solvers of the Maxwell equations like the T-matrix method^25^ and discrete dipole approximation^39^. However, the refractive index *m* of the particle is not known from its image, precluding this approach unless *m* is assumed *ad hoc*.

It becomes clear then, that what is needed is a method capable of imaging particles while simultaneously collecting their light scattering patterns all in a contact-free way. With the exception of spherical and elipsoidal particles, which constitute a minority of CMA particles^40^, this ability has not been demonstrated in a flowing aerosol stream. Here we present an experiment that does achieve this ability. With digital holography and spatial filtering, images of free-flowing particles in the CMA size range and their scattering patterns are obtained simultaneously. This “solves” the inverse problem in the sense that one is able to confidently correlate a measured pattern to the particle properties of size, shape, and orientation free of assumptions.

## Results

### Experimental design

**Figure 1:**
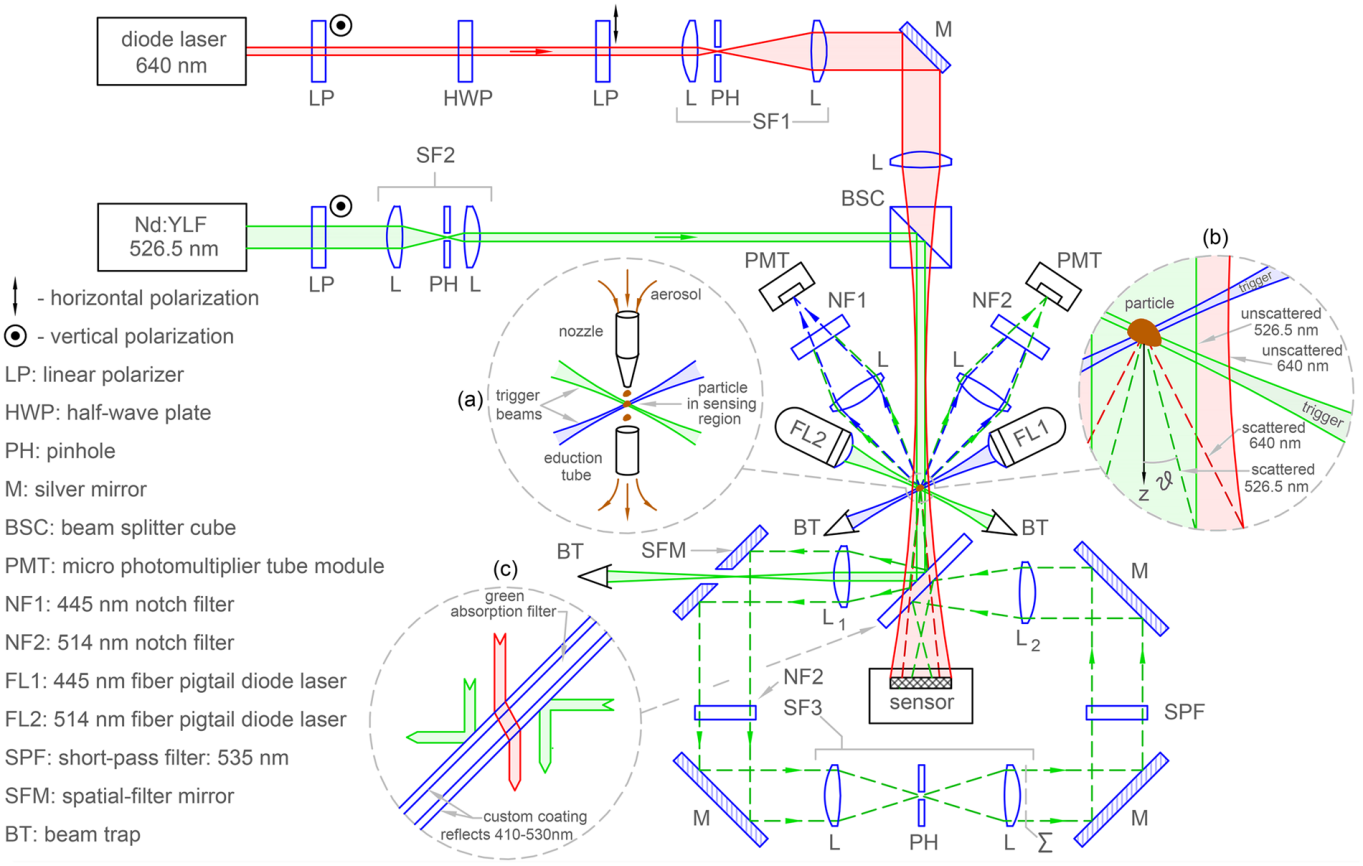
Experimental arrangement. Free-flowing aerosol particles from a nozzle pass through the intersection of a crossed-beam optical trigger (**a**) monitored by two PMTs. A trigger event corresponds to coincidence of the PMT module signals, which activates a single pulse emission from the red (640 nm) and green (526.5 nm) lasers. These beams are combined and illuminate the particle, see (**b**). Scattered light is indicated by dashed lines and unscattered by solid lines. To limit beam interference before interaction with the particle, the (linear) polarization of the red beam is rotated 90° with respect to the (linear) polarization of the green beam by a half waveplate. After scattering from the particle, red and green light are separated by the composite filter (**c**). Red light is passed directly to the sensor while green light is reflected. Following removal of the unscattered green light by the SFM, stray light and scattering from ambient dust are removed from the particle’s scattered light by spatial filter SF3. Lens L_2_ then images the output plane of SF3, labeled Σ, onto the sensor via reflection from the back side of the composite filter.

The optical arrangement begins with an aerosol nozzle that delivers a stream of particles to a sensing region. Here, the particles pass through the intersection of two trigger beams^41^ with wavelengths 445 and 515 nm. Monitoring this intersection are two micro-photomultiplier tube (PMT) modules. One PMT is guarded by a 445 nm notch filter and the other by a 514 nm filter. If a flowing particle enters the intersection, both wavelengths of light are scattered simultaneously resulting in a coincidence of the PMT signals. The particles are then removed by an eduction tube, see Fig. 1a. A logic AND gate processes the PMT signals (see Methods) to produce a trigger that queues a single-pulse emission from two additional lasers. One of these is a 640 nm diode laser, called “red” light for short; the other is a Q-switched Nd:YLF laser, second harmonic 526.5 nm, or “green” light for short. The pulse widths are approximately 150 ns and 30 ns for the red and green lasers, respectively. In either case, these pulses are short enough given the flow rate of the aerosol (~1 m/s) to prevent blurring of the subsequent measurements from particle motion.

The red and green pulses are directed to the particle as follows. First, to reduce interference effects between the pulses, the red beam is passed through a half-wave plate to rotate its polarization 90° with respect to the green beam. The red-beam profile is cleaned and expanded to one cm in diameter by a pinhole spatial filter, see SF1 in Fig. 1. The green beam is also cleaned by a spatial filter (SF2), but contracted to one mm in diameter. The red beam is then focused to a waist approximately one cm before reaching the particle and is combined with the green beam by a beam-splitting cube. A portion of the red and green light scatter from the particle while the majority of the light is unscattered, see Fig. 1b. Next, both scattered and unscattered light encounter a composite filter consisting of a dichroic coating on a green absorption filter. The coating reflects green light while passing red light, see Fig. 1c. Any (small) portion of the green light that passes the coating is absorbed by the filter substrate. Thus, both scattered and unscattered red light pass the filter to reach a colour sensor located approximately six cm from the particle. Here the waves interfere to produce an in-line, or Gabor-type, hologram of the particle. The reason that the red beam is focused before the particle is to increase the light intensity at the particle while also allowing interference between scattered and unscattered light across most of the sensor surface.

Meanwhile the scattered, and more intense unscattered, green light reflected by the composite filter is intercepted by a positive lens L_1_. The focal length of L_1_ is six cm, which is the same as the optical path- length from the lens to the particle via reflection from the composite filter. Consequently, the unscattered (collimated) light is focused by L_1_ to a waist in its back focal plane while the scattered (diverging) light is collimated. At the waist, there is a mirror with a 500 μm diameter through-hole oriented at 45° to the mirror surface. Thus, the intense unscattered light passes through the hole while the scattered light is reflected. In this way, the mirror spatially filters the light reaching lens L_1_, and as such, is called a spatial filtering mirror (SFM) in the following. Next, stray-light noise originating from ambient dust is removed from the particle’s scattered light by another spatial filter (SF3). Any stray trigger-beam and hologram-beam (red) light is removed by 514 nm and 535 nm notch and short-pass filters, respectively. Finally, the scattered light is imaged onto the sensor by lens L_2_ via reflection from the backside of the composite filter.

The sensor records the particle’s “raw” hologram in the red channel and the scattering pattern in the green channel *simultaneously*. The hologram is termed raw as it will later be modified by a background subtraction process. Taking the origin of a spherical polar coordinate system to be at the particle with the positive z-axis along the forward-scattering direction as shown in Fig. 1b, the sensor records the scattering pattern as a function of polar scattering angle *θ* and azimuthal angle *ϕ*, i.e., *I* ^sca^(*θ*, *ϕ*). Because L_1_ is one focal length from the particle, the Abbé sine condition links the sensor pixel-number in the scattering pattern to *θ* ^42^. By subtracting a particle-free background measurement from the raw hologram, a contrast hologram is formed, which improves the subsequent image quality^43^. A silhouette-like image of the particle can then be rendered from the contrast hologram by application of the Fresnel-Kirchhoff diffraction integral^43–46^ (see Methods). The image may then be directly compared to the scattering pattern revealing how the size, shape, and orientation of the particle correlate with features in the pattern. Because particle flow from the nozzle is not controlled, the position of a particle is only known to within the spatial overlap of the trigger beams, which is approximately 500 μm. A post-measurement autofocus procedure^47, 48^ is then used when rendering particle images from the hologram so that precise knowledge of the particle position is not needed prior to the measurement.

**Figure 2:**
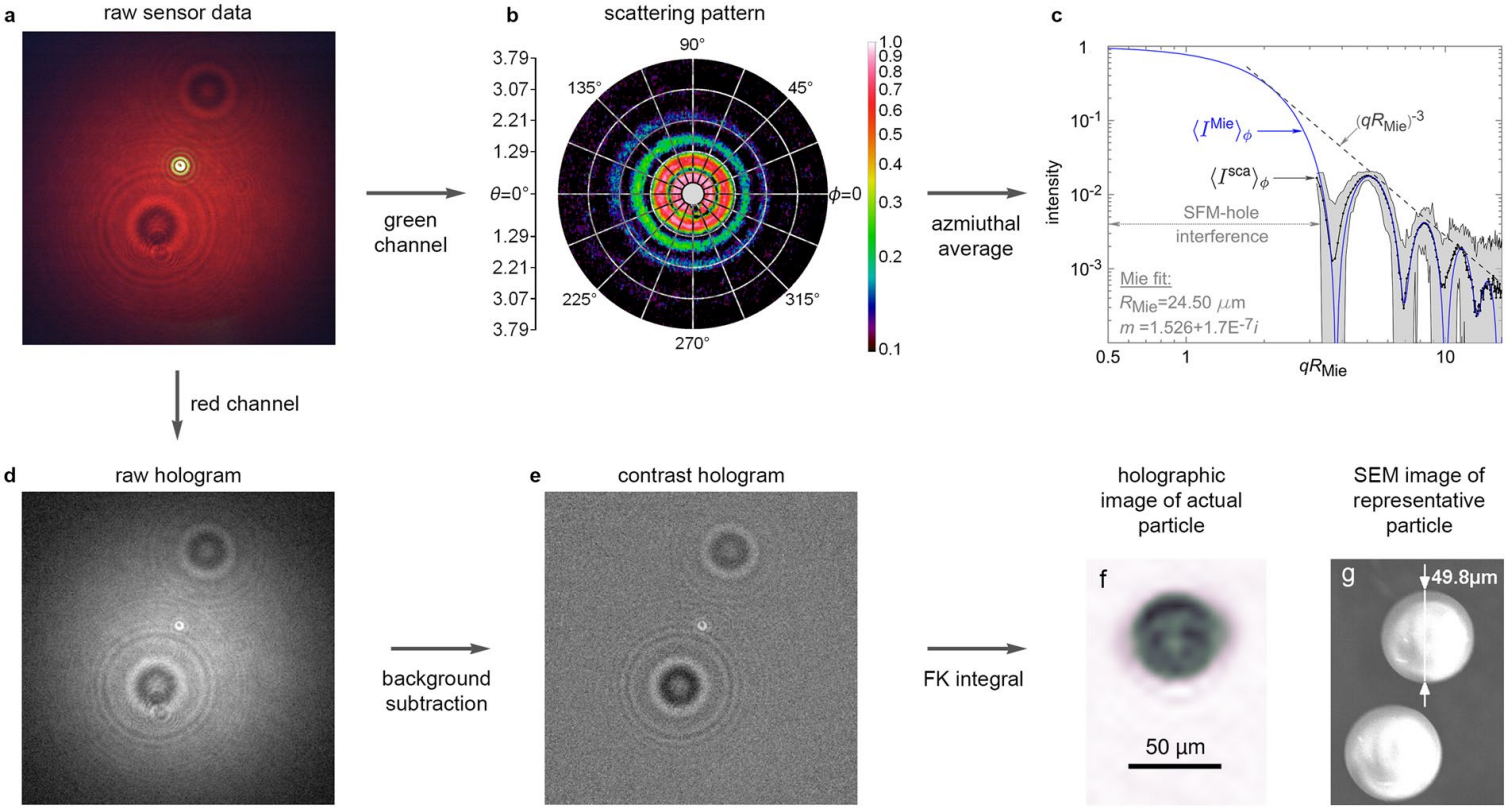
Spherical aerosol particle. Image (**a**) shows the raw sensor output for a single 50 μm diameter silica glass microsphere aerosol particle activating the optical trigger system in Fig. 1a. The green channel of this sensor data constitutes the particle’s scattering pattern, *I* ^sca^(*θ*, *ϕ*). By overlaying a polar-coordinate grid (*θ*, *ϕ*) where *θ* is measured along radials of the grid and *ϕ* around the concentric rings, (**b**) displays the pattern in false-colour in log scale. The nested-ring structure observed is emblematic of spherical-particle scattering patterns. Azimuthal averaging of this pattern gives the scattering curve (**c**) shown in log-log scale as a function of *qR*_Mie_ (see text and Methods). The gray-shaded region shows the maximum and minimum extremes of the curve for the individual values of *ϕ* considered in the average. Selecting the red channel of the sensor isolates the particle’s raw digital hologram (**d**). After forming the contrast hologram (**e**), the Fresnel-Kirchhoff (FK) operation (see Methods) is evaluated to produce an image of the aerosol particle (**f**). The scale bar is determined by the calibration procedure in Methods. In (**g**) is shown an SEM image of similar particles taken from the powder-stock used to produce the aerosol. The small disk-like feature at the center of (**d**) and (**e**) is the green light of the scattering pattern that is not completely blocked by the sensor’s pixel-level filters from entering the red channel. This feature appears to have no noticeable impact on the particle image due to the comparatively small region of the hologram it occupies.

A first example is presented in Fig. 2 for a spherical aerosol-particle. Here, the raw sensor data (a) is separated into the scattering pattern (b) and raw hologram (d). The contrast hologram (e) is then formed as described above from which the particle image (f) is derived. For comparison, a scanning electron microscope (SEM) image of a similar particle taken from the aerosol source powder is shown in (g). The scattering pattern displays the classic nested-ring structure and strong decay with increasing *θ*. A small portion of the central region of the pattern is lost from the through-hole in the SFM, and approximately $0.40^\circ \mathop{ < }\limits_{ \tilde {}}\theta \mathop{ < }\limits_{ \tilde {}}3.79^\circ$ of the scattering pattern is resolved over all azimuthal angles, i.e, 0° ≤ *ϕ* < 360°. A mapping from sensor pixel-number to scattering angles (*θ*, *ϕ*) and physical length scales in the holographic image is achieved by a calibration procedure summarized in the Methods section. Averaging the scattering pattern in (b) over the azimuthal angle *ϕ* yields the scattering curve, 〈*I* ^sca^(*θ*)〉_*ϕ*_ in (c). Here, the curve is presented as a function of the dimensionless quantity *qR*_Mie_ = 2*kR*_Mie _sin (*θ*/2), where *q* is the scattering wavevector, *k* = 2*π*/*λ* (for the green beam), and *R*_Mie_ is the particle radius as determined by comparison to Mie theory, see Methods. Plotting the curve in this way reveals power-law trends, which are often useful^26, 27, 29^ to infer particle characteristics from such scattering patterns. Here, one can see the (*qR*_Mie_)^−3^ power law for example^49^.

Inspection of the particle image in Fig. 2f reveals a fuzzy halo around the silhouette. This is due to the out-of-focus twin image^44^ inherent in Gabor-type holography and the limited resolution of the imaging process. From the wavelength of the (red) hologram beam, the geometry of the optical arrangement, and the pixel size and array size of the sensor (3.1 μm × 3.1 μm pixel size, 4240 × 2824 array), a theoretical image resolution^44^ of ~3.6 μm is possible. However, this is the case only if the whole sensor array resolves the hologram interference pattern. Due to the sensor’s limited dynamic range, which is 8-bits per channel, only a fraction of the array resolves this pattern. For most particles considered, this degrades the resolution to ~17 μm but can be improved with higher dynamic-range sensors.

### Survey of coarse-mode particles

**Figure 3:**
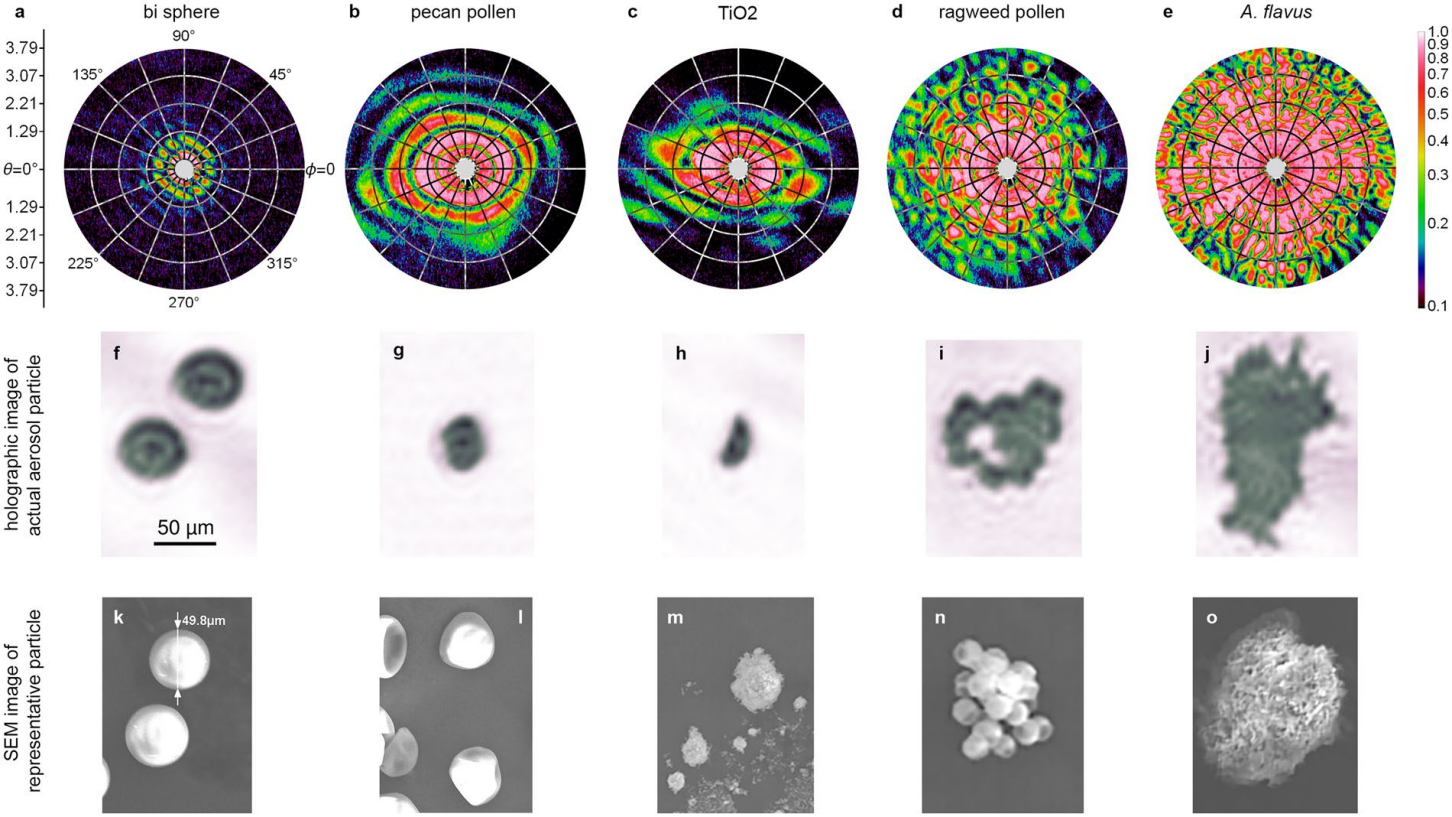
Particle survey. The top row (**a**–**e**) displays the scattering patterns *I* ^sca^(*θ*, *ϕ*) measured for aerosol particles generated from a variety of dry powders. These include 50 μm diameter silica glass spheres (**a**), a pecan pollen grain (**b**), TiO_2_ powder (**c**), ragweed pollen grains (**d**), and an *A*. *flavus* propagule (**e**). The same polar-coordinate grid and false-colour intensity log-scale is used as in Fig. 2. Notice that from the patterns alone, it is not obvious what is the size and shape of the particles. In the second row (**f**)–(**j**) are the holographic particle images of each aerosol particle in the orientation that the particle takes when the pattern is measured. The scale bar is 50 μm in (**f**) and applies to all particle images. The last row (**k**)–(**o**) displays SEM images of similar particles taken from the powders used to produce the aerosols. Thus, these SEM images are not those of the particle producing the scattering patterns or holographic images, but do provide a general sense for the variability of size and shape in the particles.

To explore the capabilities of this method, a variety of more-complex dry-powder particles are considered. These include silica glass microspheres (for calibration purposes), pecan pollen, TiO_2_ powder (MD proxy), ragweed pollen (an allergen), and *Aspergillus flavus* propagules (agricultural-crop disease pathogen^50^). The results of the experiment are presented in Fig. 3. Each column displays, respectively, the scattering pattern, holographic particle image, and SEM image of a representative particle taken from the aerosol source powder. Note that for simplicity in the experiment the pollen particles used are dead, dried particles that do not display the same morphology of living pollen. Living pollen could be investigated with this method, however, by using live catkins as the aerosol source.

Comparing the patterns and particle images in Fig. 3 reveals several details of note. Figure 3a is a pattern characteristic of a spherical particle but modulated by a linear fringe structure. The holographic image, Fig. 3f, reveals that the particle is in fact two particles, a separated bi-sphere with a sphere centre-to-centre separation of approximately Δ*d* = 70 μm. The linear structure in the pattern has an angular spacing of approximately ${\rm{\Delta }}\theta \sim 0.48^\circ$, which is consistent with the expectation from Young’s double-slit experiment of ${\rm{\Delta }}\theta =\lambda /{\rm{\Delta }}d\sim 0.43^\circ$. Note that the orientation of the linear structure is also consistent with the orientation of the bi-sphere. Lastly, the fidelity of the ring-like component of the pattern suggests that the two particles are nearly equal in size, which again, is consistent with the holographic image.

The remaining examples in Fig. 3 present particles of various size and increasing morphological complexity. Comparing the scattering patterns in Fig. 3b–e to their corresponding holographic particle images, Fig. 3g–j, shows that as the degree of sphericity of the particle degrades, the patterns evolve from a nested-ring structure to a complex speckle-like pattern. In particular, the angular size of the speckle is (qualitatively) inversely related to the size of the particle. This gives credence to the idea^37, 51^ that such speckle features, and the related concept of scattering-pattern entropy^52^, may be useful to estimate particle size and surface roughness. Perhaps most salient is to illustrate how the method is a solution to the inverse problem. The last two particles in Fig. 3 are clusters of ragweed pollen grains and an *A*. *flavus* propagule. The holographic images suggests that the particle producing the pattern in (d) is a cluster of sphere-like components roughly 20 μm in diameter, while that in (e) consists of smaller more irregular components. Knowing that individual ragweed pollen grains are sphere-like and typically 20–30 μm in size^53^, one can discriminate the scattering patterns. Attempting to do so with the scattering patterns alone would be difficult since the added complexity of the pattern in (e), compared to (d), could not confidently be attributed to any specific feature of the particle cluster without additional information. Also important here is that with both the scattering pattern and particle image in hand, one could test the efficacy of existing methods to infer particle characteristics directly from patterns.

## Discussion

This work is not the first to image aerosol particles and collect their scattering patterns, but is to our knowledge, the first to do so on free-flowing *arbitrarily* shaped particles. In the previous work, a particle is either held in an electrodynamic^33–35^ or optical trap^36, 37^ and imaged with a telemicroscope while the scattering pattern is collected. Similar measurements have also been done on an ensemble of flowing liquid drops produced with highly uniform size^38^. In the former case, the measurements are essentially equivalent to that in Fig. 3. Yet, because a telemicroscope is used, a well focused image is only possible because trapping confines the particle within the fixed focal-depth of the image sensor. In addition, that approach requires the use of two sensors; one for the image and another for the scattering pattern. Thus, the approach would encounter difficulties if multiple particles were present as they would only be well imaged if concentrated into a small trapping volume within the depth-of-focus.

In the latter case–the repeated droplet measurement–the particle images are the average of successive particles produced from a droplet-on-demand generator. This approach is successful because the generator creates drops so regular in morphology^38^ that their scattering patterns are nearly identical. Thus, the measured pattern of a single droplet can plausibly be attributed to the particle morphology resulting from an ensemble average of multiple single-particle images. Here too, the particles must be well confined in their trajectory from the droplet generator so that in-focus images are obtained. With the holographic method, the (computational) after-the-fact focusing ability relieves one from the need to trap or tightly control the flow of particles. And, if multiple particles happen to be present in the sensing region during the measurement, each can be brought into focus one-by-one at a later time from a *single* hologram measurement. This ability can be used to approximate the three-dimensional structure of a single particle^48^.

One can also extract useful information from the hologram directly. In recent work^54, 55^, we demonstrate that a particle’s extinction cross section *C* ^ext^ can be estimated by simple integration of the contrast hologram. Moreover, the *C* ^ext^ value is often less than 5% in error^54^ for either spherical or nonspherical CMA particles. In the case of optically large particles, it is possible that the scattering cross section *C* ^sca^ may also be estimated from the measured scattering pattern. That is, since such large particles scatter light most strongly around the forward direction (*θ*, *q* = 0), and do so typically by orders of magnitude compared to larger angles beyond the Guinier range^56, 57^ of $q\mathop{ > }\limits_{ \tilde {}}1/R$, integration of the scattering pattern could be expected to yield *C* ^sca^ with good accuracy. Although the range of errors involved with this estimation is a topic of current study, the prospect is that if *C* ^ext^ and *C* ^sca^ may be retrieved, one could estimate the single-particle absorption cross section as *C* ^abs^ = *C* ^ext^ − *C* ^sca^. This would have major implications for atmospheric aerosol studies as it would enable the first *in situ* measurements of the single-scattering albedo *ϖ*, an important quantity in climate models. Moreover, by adding particle illumination in the sensor’s blue channel, it may be possible to gain dispersion information by comparing the red and blue-channel scattering patterns. It is for these reasons, in addition to the imaging and scattering pattern measurement ability, that we claim that the two-colour holographic approach effectively solves the inverse problem; a near complete make-up of a *single* particle’s morphology and scattering is potentially revealed from a *single* measurement on a *single* sensor.

However, there are particle characteristics not revealed in this approach that prevent it from constituting a complete solution to the inverse problem. Foremost is that the refractive index is not provided by the holographic image. What is not tested in our work to date is whether the scattered wave-phase information that is available from the hologram (see Methods) could provide information useful in this regard. Refractive index profiles for stationary transparent particles can be obtained in this way^43^ and may extend to the more complex particles considered here. The material phase of the particle^58^ is also of interest in atmospheric science contexts, e.g., to differentiate liquid and solid components in cloud-water particles. Elegant work on trapped spherical particles shows^59^ that signatures of such phase-change do appear in the scattering patterns. It is conceivable that these same techniques could be integrated with the approach here, thus enabling detection of phase changes in arbitrary particles.

## Methods

### Aerosol production

The aerosols are produced from several grams of dry powder placed in a 250 ml chamber under positive pressure. A magnetic stir-bar agitates the powder leading to suspended single and multi-particles clusters. These are carried from the chamber along silicone tubing to an aerosol nozzle. The particles emerge from the nozzle in a jet-like spray into the sensing region in Fig. 1a. Pressure in the generation chamber is varied to control the particle flow-rate. To prevent cross-contamination of the particle samples, the tubing and nozzle are replaced from one particle type to another and the chamber is cleaned.

### Trigger

To produce the queue signal that activates the red and green lasers when a particle is in the sensing region, the PMT signals are processed as follows^60^. First, each PMT signal is fed to a dedicated amplifier unit (ORTEC 570) set for 1000× gain. The amplified signals are then passed to an analyser (ORTEC 850) to apply a squelch level. This is needed to prevent trigger events by particles that are too small to be resolved, i.e., sub-micron particles. Following the analyser, the signals are passed to an analogue processor (SRS) giving a strong rising edge to each signal transient. Lastly, the signals are passed to an AND logic gate (ORTEC CO4042). If the signals are in coincidence, indicating the presence of a particle at the trigger-beam intersection, a positive TTL pulse is sent to a digital delay generator (SRS DG645) controlling the red (Coherent, Inc. OBIS LX) and green (Photonics Industries, Inc. DC50-527) lasers. The delay generator allows adjustment of the red laser pulse’s (temporal) length; ~150 ns is used here. Alignment of the trigger beams is achieved by inserting an optical fibre in the aerosol nozzle outlet such that the fibre follows the trajectory of the aerosol particles (the aerosol is not present during this procedure). With the trigger laser units mounted to three-axis translation stages, each beam is positioned so that they scatter from the same portion of the fibre. This scattered light is also used to align the PMT modules.

### Holographic image formation

The red channel of the image-sensor (Point Grey Research Inc. GS3-U3-120S6C-C) resolves the (intensity) interference pattern *I* ^holo^ produced by the incident and forward-scattered light from the particle. An image is later rendered from *I* ^holo^ by computationally treating this hologram as a transmission diffraction-grating illuminated by the same incident light, which is approximated as a plane wave across the particle. This is permissible because the particle is much smaller than the red-beam waist. Here, the diffraction process is modelled approximately with the Fresnel-Kirchhoff scalar-wave diffraction theory under the Fresnel approximation, e.g. see Eq. (3.38) in Kreis^44^. Prior to evaluation of this Fresnel-Kirchhoff (FK) operation, *I* ^holo^ is subtracted from the particle-free background, or reference intensity *I* ^ref^, to yield a contrast hologram *I* ^con^ = *I* ^ref^ − *I* ^holo^. In practice, this subtraction is important as it removes most imperfections in the illumination-beam profile, stray-light noise, and improves the subsequent particle image. Application of the FK operation to *I* ^con^ produces a complex-valued function *K*(*z*) that can be thought of an approximation of the particle’s near-field scattered wave amplitude at a distance *z* from the hologram^43–46^. Thus, evaluation of |*K*(*z*)|^2^ provides an image of the particle if *z* corresponds to the “focus distance” for the imaging process^47, 48^. Note that one is not obliged to take the absolute square, in which case scattered-wave phase information becomes available.

### Calibration

**Figure 4:**
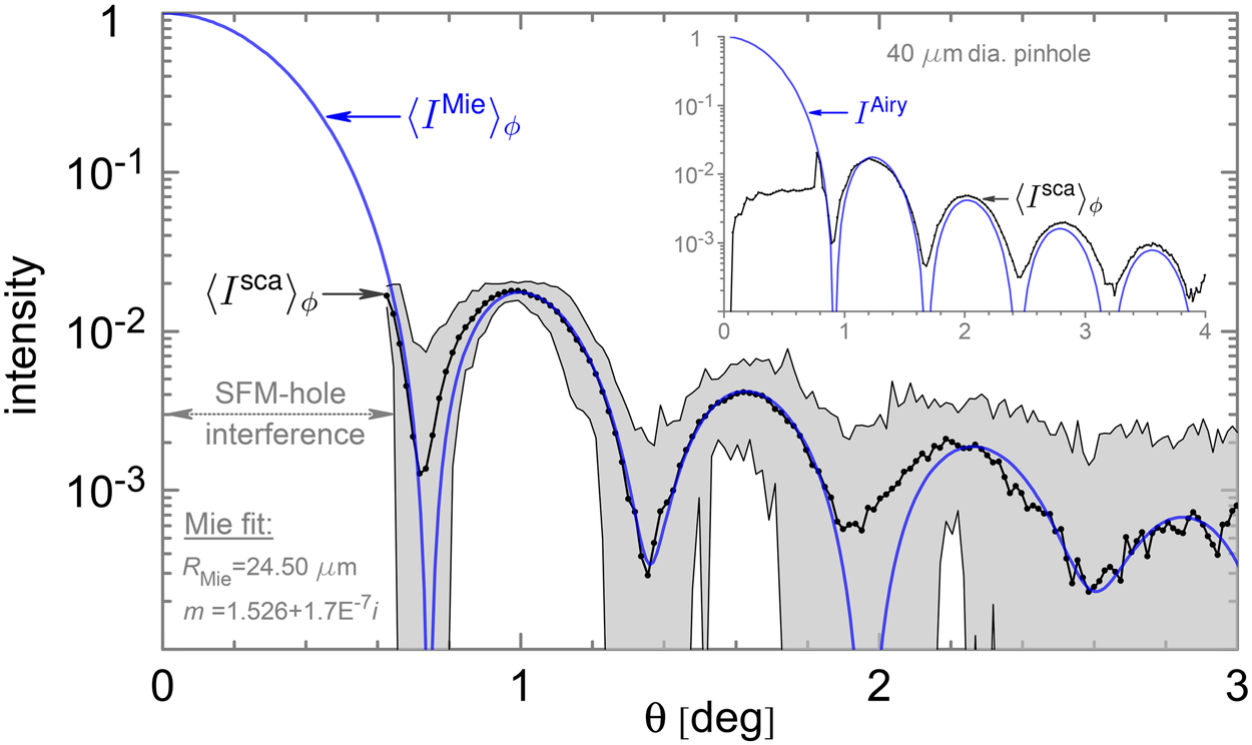
Calibration. The inset shows comparison of the Airy diffraction pattern *I* ^Airy^ calculated for a 40 μm diameter pinhole and the measured pattern from such a pinhole placed at the aerosol nozzle outlet in Fig. 1. The measured diffraction pattern is averaged over the azimuthal angle, i.e., 〈*I* ^sca^〉_*ϕ*_. The erratic behaviour in *I* ^Airy^ over the angular range $0^\circ \le \theta \mathop{ < }\limits_{ \tilde {}}0.8^\circ$ is due to the interference of the hole in the SFM. The main plot presents the comparison of the azimuthally averaged scattering pattern 〈*I* ^sca^〉_*ϕ*_ for the same single spherical aerosol particle of 50 μm diameter in Fig. 2. Mie theory is then used to generate a simulated scattering curve 〈*I* ^Mie^〉_*ϕ*_ that is fit 〈*I* ^sca^〉_*ϕ*_ by varying the sphere radius *R*_Mie_ in Mie theory. The fit value for *R*_Mie_ agrees well the manufacturer provided mean particle-size as shown. The gray-shaded region indicates the maximum and minimum extremes of the scattering curve for the individual values of *ϕ* considered in the azimuthal average.

Translation of sensor pixels to scattering angles (*θ*, *ϕ*) is achieved by replacing the aerosol nozzle with a 40 μm diameter pinhole. The measured diffraction pattern is then fit to the Airy function *I* ^Airy^ using free parameters *α*, *β*, and *γ* as follows. First, the centre of the measured pattern *I* ^sca^ is found from the first diffraction minimum. This centre-point corresponds to the forward scattering direction (*θ* = 0°) and is within the missing portion of the pattern lost from the hole in the SFM (Lenox Laser Inc., AL-45-500-CUST-2″). With the forward direction identified, the pattern is then averaged over the azimuthal *ϕ* angle. This yields a scattering curve 〈*I* ^sca^〉_*ϕ*_ as a function of radial distance *r* in pixels from *θ* = 0° to the edge of the sensor. To translate *r* into *θ*, the Abbé sine condition^42^ *r* = *f* sin *θ* is used, where *f* is the focal length of lens L_1_ in Fig. 1. However, it is observed that the diffraction pattern displays a slight radial distortion likely due to aberration from L_1_. To correct^61^ for this, a power-law scaling of *θ* is made as *γθ* − *βθ* ^2^. Finally, with *α* used to scale the overall magnitude of 〈*I* ^sca^〉_*ϕ*_, the parameters *α*, *β*, and *γ* are adjusted to bring 〈*I* ^sca^〉_*ϕ*_ into best agreement with *I* ^Airy^. The result can be seen in the Fig. 4 inset. The same values of *α*, *β*, and *γ* are then used in the remainder of the study. The effectiveness of these three calibration parameters is tested with 〈*I* ^sca^〉_*ϕ*_ for the spherical aerosol particle (Fig. 1) by comparison to Mie theory in Fig. 4. Here, a refractive index^62^ of *m* = 1.526 + 1.7E^−7^ *i* at 526.5 nm is used in the Mie calculation and the particle radius *R*_Mie_ is then varied to best match 〈*I* ^Mie^〉_*ϕ*_ to 〈*I* ^sca^〉_*ϕ*_. The procedure yields *R*_Mie_ = 24.50 μm in excellent agreement with the manufacturer’s value of *R* = 24.5 ± 0.70 μm, although some discrepancy is seen between the curves in Fig. 4. This process also allows for the length scales in the holographic particle images to be calibrated to true physical lengths. Close inspection of Fig. 4 shows that the SFM hole strongly affects the scattering curve from *θ* = 0° to *θ* ~ 0.65° while the two-dimensional (2D) scattering patterns in Figs 2b and 3a–e show loss of the pattern only up to *θ* ~ 0.40°. The reason for this apparent discrepancy is that the SFM hole is not precisely centred on the *θ* = 0 direction. Thus, when the pattern is azimuthally averaged (Fig. 4), the resulting curve shows an apparent larger angular effect due to the off-centre SFM hole as compared to the 2D patterns.

### Data availability

The data supporting the findings of this study are available within the article and upon request made to M.J.B.
